# *Mycobacterium cosmeticum*, Ohio and Venezuela

**DOI:** 10.3201/eid1308.061061

**Published:** 2007-08

**Authors:** Robert C. Cooksey, Jacobus H. de Waard, Mitchell A. Yakrus, Sean R. Toney, Omaira Da Mata, Scott Nowicki, Kevin Sohner, Elizabeth Koch, Cathy A. Petti, Roger E. Morey, Arjun Srinivasan

**Affiliations:** * Centers for Disease Control and Prevention, Atlanta, Georgia, USA; † Instituto de Biomedicina, Caracas, Venezuela; ‡ Ohio Department of Health, Columbus, Ohio, USA; § ARUP Laboratories, Salt Lake City, Utah, USA

**Keywords:** Mycobacterium cosmeticum, catheter infection, rapidly growing mycobacteria, letter

**To the Editor:** *Mycobacterium cosmeticum* is a rapidly growing nontuberculous mycobacteria species that was first described in November 2004. The first strains were obtained from cultures of a sink drain in a nail salon in Atlanta, Georgia, USA, and from a granulomatous lesion of a female mesotherapy patient in Venezuela (*1*).

Among 3 additional isolates of *M. cosmeticum* obtained from July 2003 through November 2004, one was obtained from a 77-year-old man who was admitted to Ohio hospital A on September 22, 2004, with fever, exacerbation of chronic obstructive pulmonary disease, and urosepsis. Underlying medical conditions included diabetes, discitis, hyperlipidemia, coronary artery disease, and coal worker’s pneumoconiosis. He had received intravenous antimicrobial agents (rifampin and daptomycin) through a Groshong catheter that had been inserted to treat discitis. A routine blood culture was performed according to standard methods (*2*), and the catheter was removed. A diagnosis of catheter-associated bacteremia (CAB) was made, but the patient’s overall condition improved without antibacterial drug therapy, and he was discharged 4 days after admission. The culture specimen yielded only mycobacteria and was sent on to ARUP Laboratories, where it was identified as *M. cosmeticum* by 16S rDNA sequence analysis. The isolate was then sent to the Centers for Disease Control and Prevention (CDC) Mycobacteriology Laboratory Branch (Atlanta, GA, USA) and designated OH1.

A 43-year-old woman with a diagnosis of non-Hodgkin lymphoma, who had received regular central venous catheterizations, was admitted to Ohio hospital B on August 20, 2004. A left subclavian catheter was inserted, and a routine blood specimen for culture was subsequently obtained on the day of admission. Before admission, the woman had been receiving acyclovir and cefepime. She received chemotherapeutic agent injections, platelet infusion, and an autologous stem cell transplant 6 days after admission. The blood culture was positive only for rapidly growing mycobacteria, and the final diagnosis was CAB. However, no symptoms of infection were observed, and no antimycobacterial drug therapy was administered. She was discharged without complications after the transplant was received and the catheter removed. The bacterial isolate was forwarded to CDC’s Special Bacteriology Reference Laboratory, where it was identified as *M. cosmeticum* by 16S rDNA sequence analysis, sent on to the CDC Mycobacteriology Laboratory Branch, and designated OH2.

A 36-year-old man with AIDS was admitted to hospital C in Caracas, Venezuela, in June 2003 with dyspnea, fever, and expectoration. A sputum sample was positive by acid-fast bacillus smear and culture, yielding both *M. cosmeticum* (designated VZ1) and *M. scrofulaceum* on Middlebrook 7H10 agar (Remel Co., Lenexa, KS, USA). At the time the sputum was obtained, the patient was receiving only trimethoprim-sulfamethoxazole, but he experienced respiratory arrest and died ≈6 weeks later.

The 3 isolates were confirmed to be *M. cosmeticum* by high-performance liquid chromatography mycolate analyses and by PCR restriction analysis of a 440-bp segment of *hsp*65 (*1*). The relationship of these isolates to the only documented strains of *M. cosmeticum* was evaluated by analysis of large restriction fragments with pulsed-field gel electrophoresis (*1*) and by repetitive element PCR (*3*). Banding patterns for isolates OH1 and OH2 were different from one another as well as from isolate VZ1 and the 2 control strains. Typing patterns for isolate VZ1, however, matched the control strain from Venezuela (ATCC BAA-878^T^), which indicates that these 2 isolates are likely a common strain (Figure).

**Figure:**
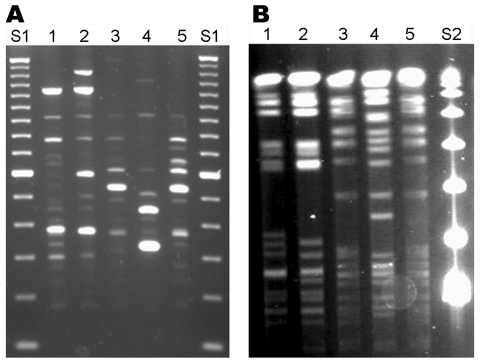
Repetitive element (Rep)–PCR (A) and pulsed-field gel electrophoresis (PFGE) (B) patterns of *Mycobacterium cosmeticum* isolates from 2 patients in Ohio and 1 patient in Venezuela. Rep-PCR was performed by using BOXA1R primer (*3*), and PFGE was performed with restriction enzyme *Ase*I. Lanes 1, 2, Ohio isolates OH1 and OH2; lanes 3, 4, control strains ATCC BAA-878^T^ and ATCC BAA-879; lane 5, Venezuelan isolate VZ1. DNA size standards are 100-bp (S1) and 48.5-kb marker (S2).

Of the >125 recognized *Mycobacterium* species, ≈50 are etiologic agents of human disease (*4*). The type strain of *M. cosmeticum* (ATCC BAA-878^T^) was associated with a soft-tissue infection in which the source was postulated to be environmental contamination of an unknown substance administered to the patient by injection as part of a weight loss regimen. This strain and isolate VZ1 were isolated from clinics in Caracas, Venezuela; both were found to be a common strain, but no other factors suggest that these represent an epidemic cluster. Although the Venezuelan patient from whom isolate VZ1 was obtained exhibited symptoms consistent with mycobacterial pulmonary disease, *M. cosmeticum* involvement cannot be proven because an additional *Mycobacterium* species, *M. scrofulaceum*, was isolated from the patient’s sputum. Because each of these organisms is found in the aqueous environment, they may represent colonization or may have been transiently present in the patient. Nonetheless, additional nontuberculous mycobacteria species have been reported to cause pulmonary disease, and the involvement of *M. cosmeticum* in this case cannot be excluded.

Successful treatment of CAB infections caused by rapidly growing mycobacteria has most often been achieved by removing the catheter with or without the use of antimicrobial drug therapy (*4*). Criteria to support a true bloodstream infection were met by one of the patients in Ohio. These criteria include the absence of a source for bacteremia alternative to *M. cosmeticum* OH1 and the resolution of the febrile syndrome after removal of the device. The second patient had no symptoms when the blood culture was obtained; thus, the clinical significance of *M. cosmeticum* in this case is unclear.

When all identified strains of *M. cosmeticum* are considered, this species is clearly present in diverse geographic regions and in healthcare institutions. These findings suggest that it may be widely distributed in the environment and should be regarded, along with other rapidly growing mycobacteria species, as a potential pathogen.
